# Co-Occurring Alcohol Use Disorder and Schizophrenia

**Published:** 2002

**Authors:** Robert E. Drake, Kim T. Mueser

**Affiliations:** Robert E. Drake, M.D., Ph.D., and Kim T. Mueser, Ph.D., are professors in the departments of psychiatry and community and family medicine at Dartmouth Medical School and the New Hampshire-Dartmouth Psychiatric Research Center, Lebanon, New Hampshire

**Keywords:** comorbidity, AODD (alcohol and other drug dependence), schizophrenia, prevalence, disease susceptibility, disease course, self-medication, reinforcement, dopamine, neurotransmission, social adjustment, treatment complications, combined modality therapy, correlation analysis, literature review

## Abstract

Alcohol use disorder (AUD) is the most common co-occurring disorder in people with schizophrenia. Both biological factors and psychosocial factors are thought to contribute to this co-occurrence. Schizophrenia patients with AUD are more likely to have social, legal, and medical problems, compared with other people with schizophrenia. AUD also complicates the course and treatment of schizophrenia.

Schizophrenia is a severe and disabling psychiatric disorder characterized by persistent delusions, hallucinations, disorganized speech, disorganized behavior, and negative symptoms such as the absence of emotional expression or a lack of motivation or initiative (American Psychiatric Association [APA] 1994). Alcohol use disorder^1^ (AUD) commonly co-occurs with schizophrenia. This article reviews several aspects of AUD among people with schizophrenia, including the prevalence of this co-occurrence, biological and psychosocial factors that contribute to this relationship, correlated problems dually diagnosed people experience, the effects of AUD on the course and outcome of schizophrenia, treatment issues, and public policy implications. People with schizophrenia and AUD frequently abuse other substances as well. Current understanding of contributing factors, correlated problems, effect on course of illness, and treatment implications is similar for different substances of abuse.

## Prevalence and Contributing Factors

Schizophrenia is frequently complicated by comorbid disorders such as medical illnesses, mental retardation, and substance abuse. Substance use disorder is the most frequent and clinically significant comorbidity in this population, and alcohol is the most common substance of abuse, other than nicotine (nicotine is much more prevalent than any other substance of abuse in this population) (Cuffel 1996). Undoubtedly, the availability of alcohol and the fact that it is legal contribute to its widespread abuse among people with schizophrenia as well as in the general population. The Epidemiologic Catchment Area (ECA) study^2^ found that 33.7 percent of people with a diagnosis of schizophrenia or schizophreniform disorder (a related disorder marked by the same symptoms as schizophrenia but lasting less than 6 months) also met the criteria for an AUD diagnosis at some time during their lives and that 47 percent met the criteria for any substance use disorder (excluding nicotine dependence) (Regier et al. 1990). Rates of substance use disorder tend to be higher among males and among people of both genders and all ages in institutional settings, such as hospitals, emergency rooms, jails, and homeless shelters. This holds true for people with and without schizophrenia (Regier et al. 1990).

The high rates of AUD and other substance use disorders in people with schizophrenia appear to be determined by a complex set of factors (described below) (Mueser et al. 1998). People with schizophrenia probably use alcohol and other drugs for many of the same reasons as others in society, but several biological, psychological, and socioenvironmental factors have been hypothesized to contribute to this population’s high rates of substance use disorders.

### Biological Factors

There are three possible biological factors. First, many clinicians and researchers have asserted that people with schizophrenia use alcohol and other drugs to self-medicate in an attempt to alleviate the symptoms of schizophrenia or the side effects of the antipsychotic medications prescribed for schizophrenia (Chambers et al. 2001). Research evidence does not strongly support this view, however. For example, alcohol abuse often precedes schizophrenia; specific drugs of abuse are not selected in relation to specific symptoms; and various substances of abuse produce a range of different effects but generally exacerbate rather than relieve symptoms of schizophrenia (Chambers et al. 2001).

Second, the underlying neuropathological abnormalities of schizophrenia (i.e., the abnormalities in the brain that characterize schizophrenia) are thought to facilitate the positive reinforcing effects of substance use (Chambers et al. 2001). A common neurological basis for schizophrenia and for the reinforcing effects of substance use may predispose people to both conditions. This common basis involves the dysregulation of the brain chemical (i.e., neurotransmitter) dopamine. This would explain why people with schizophrenia prefer drugs such as nicotine and a class of antipsychotic medications that increase dopamine transmission in some areas of the brain. Of course, the reinforcing effects of alcohol use involve multiple neurotransmitter systems, and the mechanisms at work are not yet clear (Koob and Roberts 1999). The neurobiology of schizophrenia is similarly unclear (Chambers et al. 2001).

The third hypothesis suggests that people with schizophrenia are especially vulnerable to the negative psychosocial effects of substance use because the schizophrenia syndrome produces impaired thinking and social judgment and poor impulse control. Thus, even when using relatively small amounts of psychoactive substances, these people are prone to develop significant substance-related behavioral problems that qualify them for a diagnosis of substance use disorder (Mueser et al. 1998).

### Psychological and Socioenvironmental Factors

Psychological and socioenvironmental factors also appear to contribute to the co-occurrence of schizophrenia and AUD. People with schizophrenia and AUD often report that they use alcohol and other drugs to alleviate the general dysphoria of mental illness, poverty, limited opportunities, and boredom; they also report that substance use facilitates the development of an identity and a social network (Dixon et al. 1990). An entire generation of adults with schizophrenia in the United States has grown up during the era of deinstitutionalization (Lamb and Bachrach 2001). Although residing predominantly in the community rather than in hospitals, these people still have had limited vocational, recreational, and social opportunities (caused by factors such as illness, stigma, and segregation). Further, they have experienced downward social drift into poor urban living settings, where they are regularly exposed to substance abuse and substance-abusing social networks (Lamb and Bachrach 2001).

## Correlated Problems and the Effects of AUD on the Course and Outcome of Schizophrenia

Two general types of studies of the problems experienced by people with co-occurring schizophrenia and AUD are available. The first type, cross-sectional studies, collects data at one point in time. The second type, longitudinal studies, collects data at several points over a period of time. Cross-sectional studies indicate that AUD among people with schizophrenia is associated with numerous manifestations of bad outcomes and poor quality of life (referred to generally as poor adjustment), including increased recurrence of psychiatric symptoms, psychosocial instability, other substance use disorders, violence, victimization, legal problems, medical problems such as HIV infection and hepatitis, family problems, and institutionalization in hospitals and jails (Drake and Brunette 1998). People with schizophrenia and AUD are particularly prone to unstable housing situations and homelessness. Although these people often reject medications and outpatient treatment, they nevertheless represent a high cost to the treatment system because they receive a high rate of hospital-based services—relapse, as well as familial, psychosocial, legal, housing, and other crises force them into emergency care (Dickey and Azeni 1996).

The common explanation for these correlated problems is that alcohol use causes or exacerbates poor adjustment among people with schizophrenia. Many other factors could, however, explain the relationships between AUD and poor adjustment found in cross-sectional studies. For example, schizophrenia patients who abuse alcohol often abuse other substances, fail to take medications, and live in stressful circumstances without a strong support network, as described above. They may also have other inherent differences from schizophrenia patients without AUD, thereby confounding the comparison between schizophrenia patients with AUD and those without AUD.

Researchers are also accumulating longitudinal data regarding the course and outcome of co-occurring schizophrenia and AUD. Short-term studies lasting 1 year or less of patients in traditional mental health treatment systems indicate that they are prone to negative outcomes, such as continuing alcohol abuse or dependence, high rates of homelessness, disruptive behavior, psychiatric hospitalization, victimization, and incarceration. For example, one typical study of outpatients with schizophrenia found that those with co-occurring AUD had higher rates of hospitalization and depression compared with those with schizophrenia only (Cuffel and Chase 1994).

Several studies, including some studies that tracked participants’ progress over time (rather than collecting data on patients’ histories at some later point) indicate that dually diagnosed people who become abstinent (compared with those who do not) show more positive results in other related areas, such as lower psychiatric symptoms and decreased rates of hospitalization (Drake et al. 1996). For example, people in the ECA study with schizophrenia and AUD who attained abstinence had decreased rates of depression and hospitalization at 1-year followup (Cuffel 1996). In a long-term followup study of schizophrenia patients by Drake and colleagues (1998), those who attained stable abstinence showed dramatic improvements in many domains, including decreased symptoms, decreased institutionalization, increased psychosocial stability, and self-reported improvements in quality of life. These positive findings have fueled attempts to develop more effective interventions for AUD among schizophrenia patients. As described below, such interventions include those that integrate treatment for schizophrenia and for AUD.

## Treatment

Historically, the mental health and substance abuse treatment systems in the United States have been separate, and traditional approaches to treating people with co-occurring disorders have involved parallel or sequential treatment in these separate systems. In practice, patients with co-occurring mental and substance use disorders have rarely received needed treatments (Watkins et al. 2001) and have generally experienced poor outcomes (Drake et al. 1996; Ridgely et al. 1987). As a result, there has been widespread endorsement by patients, clinicians, administrators, and researchers for integrating mental health and substance abuse services (Bellack and DiClemente 1999; Onken et al. 1997; Ries 1994). There is also accumulating research support for the effectiveness of the integrated treatment approaches that have evolved over the past two decades (Drake et al. 1998).

Integrated approaches to treatment for patients with schizophrenia and AUD are generally offered through the use of multidisciplinary treatment teams that provide outreach, comprehensive services, and stage-wise treatments (described below). Outreach is needed because these patients are often demoralized and reluctant to engage in treatment. Comprehensive services are vital because recovery involves building skills and supports to pursue a meaningful life rather than just managing symptoms or illnesses. Stage-wise treatment assumes that patients recover from two serious disorders over time, in stages, and with help from treatment providers. Patients with schizophrenia and AUD generally pass through four stages of treatment:

1. Engagement, which involves building a trusting treatment relationship
2. Persuasion, which entails developing motivation to manage both illnesses and pursue recovery
3. Active treatment, which encompasses development of the skills and supports needed for illness management and recovery
4. Relapse prevention, which involves strategies to avoid and minimize the effects of relapses (Osher and Kofoed 1989).

Although the need to provide integrated, multidisciplinary services is clear, the numerous specific treatments that are in use or in development need to be tested regarding their individual effectiveness and their effectiveness in combination (Drake et al. 2001). For example, specific individual, group, family, and self-help approaches to integrated treatment are described in the literature, but few studies validate or compare these different approaches. Similar comments pertain to potential psychopharmacologic treatments and to approaches to psychiatric rehabilitation. Several retrospective studies indicate that the antipsychotic medication clozapine may be particularly helpful to patients with schizophrenia and AUD, but the mechanisms of action for the effects on both illnesses are unclear, and controlled research is needed to establish the efficacy and effectiveness of this treatment (Green et al. 1999).

Several approaches to housing, social skills training, vocational services, money management, and supervision have also been recommended but not rigorously tested. Another important area of investigation is treatment for those patients who do not respond to standard outpatient approaches. Clinicians need to know which patients should be offered residential treatments, contingency management (i.e., providing positive consequences for desired behaviors and withholding those consequences or providing negative consequences for undesired behaviors), adjunctive medications, money management, or other second-line interventions (i.e., interventions for patients who do not respond to standard treatment) (Drake et al. 2001).

## Public Policy

Although the testing and refinement of specific interventions, the development of treatment matching, and strategies to overcome nonresponsiveness are important issues, progress toward integrating mental health and substance abuse services has been minimal. Barriers exist at all levels (e.g., organizational, financial, and educational) and public policy at the Federal, State, regional, and local levels has thus far failed to promote widespread adoption of either integrated treatments for dual disorders or other evidence-based practices in the mental health and substance abuse treatment systems (Goldman et al. 2001). Clinicians, patients, and family members can advocate for effective services, but training and even successful demonstration programs will not be sustainable if policymakers do not eliminate restrictions and provide the incentives and reinforcements for evidence-based practices.

## Summary and Conclusions

AUD and other substance use disorders are extremely common among patients with schizophrenia. Almost half of schizophrenia patients have a substance use disorder (when nicotine dependence is excluded) during their lifetime. The rate is probably even greater among high-risk groups, such as young men with a history of violence or homelessness, and among patients in acute care settings. In most geographic areas, alcohol is the most common substance of abuse (other than nicotine) among patients with schizophrenia, and alcohol abuse is correlated with poor concurrent adjustment and predictive of adverse outcomes such as higher rates of homelessness, hospitalization, and incarceration.

Current research indicates that integrating mental health and substance abuse treatments is more effective than offering services in parallel systems. Integrated treatments, generally delivered by multidisciplinary teams, emphasize outreach, comprehensiveness, and a stage-wise approach to treatment and recovery. Overcoming the barriers to the implementation of integrated treatments in routine health care settings is an immediate challenge. Specific psychosocial and pharmacological interventions also need further development and testing, particularly for patients who do not respond to basic integrated interventions.

## Footnotes

1 The term “alcohol use disorder” in this article refers to the disorder defined by criteria for alcohol abuse or dependence in the American Psychiatric Association’s *Diagnostic and Statistical Manual of Mental Disorders, Fourth Edition* (DSM–IV) (APA 1994). The terms “alcohol use disorder” and “alcohol abuse” are used interchangeably in this article. The definitions for these terms vary among studies reviewed and are frequently based on earlier versions of the DSM.

2 The ECA study was a nationwide survey that used DSM–IV criteria to determine the prevalence of psychiatric disorders in the general population and among people in treatment.

## References

[b1-99-102] American Psychiatric Association (APA) (1994). Diagnostic and Statistical Manual of Mental Disorders.

[b2-99-102] Bellack AS, DiClemente CC (1999). Treating substance abuse among patients with schizophrenia. Psychiatric Services.

[b3-99-102] Chambers A, Krystal JH, Self DW (2001). A neurobiological basis for substance abuse comorbidity in schizophrenia. Biological Psychiatry.

[b4-99-102] Cuffel BJ, Drake RE, Mueser KT (1996). Comorbid substance use disorder: Prevalence, patterns of use, and course. Dual Diagnosis of Major Mental Illness and Substance Disorder: Recent Research and Clinical Implications.

[b5-99-102] Cuffel BJ, Chase P (1994). Remission and relapse of substance use disorder in schizophrenia: Results of a one-year prospective study. Journal of Nervous and Mental Disease.

[b6-99-102] Dickey B, Azeni H (1996). Persons with dual diagnosis of substance abuse and major mental illness: Their excess costs of psychiatric care. American Journal of Public Health.

[b7-99-102] Dixon L, Haas G, Weiden P (1990). Acute effects of drug abuse in schizophrenic patients: Clinical observations and patients’ self-reports. Schizophrenia Bulletin.

[b8-99-102] Drake RE, Brunette MF, Galanter M (1998). Complications of severe mental illness related to alcohol and other drug use disorders. Recent Developments in Alcoholism. Volume 14. Consequences of Alcoholism.

[b9-99-102] Drake RE, Mueser KT, Clark RE, Wallach MA (1996). The natural history of substance disorder in persons with severe mental illness. American Journal of Orthopsychiatry.

[b10-99-102] Drake RE, Mercer-McFadden C, Mueser KT (1998). A review of integrated mental health and substance abuse treatment for patients with dual disorders. Schizophrenia Bulletin.

[b11-99-102] Drake RE, Essock SM, Shaner A (2001). Implementing dual diagnosis services for clients with severe mental illness. Psychiatric Services.

[b12-99-102] Goldman HH, Ganju V, Drake RE (2001). Policy implications for implementing evidence-based practices. Psychiatric Services.

[b13-99-102] Green AI, Strous RD, Zimmet SV, Schildkraut JJ (1999). Clozapine for co-morbid substance use disorder and schizophrenia: Do patients with schizophrenia have a reward deficiency syndrome that can be ameliorated by clozapine?. Harvard Review of Psychiatry.

[b14-99-102] Koob GF, Roberts AJ (1999). Brain reward circuits in alcoholism. CNS Spectrums.

[b15-99-102] Lamb HR, Bachrach L (2001). Some perspectives on deinstitutionalization. Psychiatric Services.

[b16-99-102] Mueser KT, Drake RE, Wallach MA (1998). Dual diagnosis: A review of etiological theories. Addictive Behaviors.

[b17-99-102] Onken LS, Blaine JD, Genser S, Horton AMS (1997). Treatment of Drug-Dependent Individuals with Comorbid Mental Disorders.

[b18-99-102] Osher FC, Kofoed LL (1989). Treatment of patients with psychiatric and psychoactive substance abuse disorders. Hospital and Community Psychiatry.

[b19-99-102] Regier DA, Farmer ME, Rae DS (1990). Comorbidity of mental disorders with alcohol and other drug abuse: Results from the Epidemiologic Catchment Area (ECA) study. JAMA: Journal of the American Medical Association.

[b20-99-102] Ridgely MS, Osher FC, Talbott JA (1987). Chronic Mentally Ill Young Adults with Substance Abuse Problems: Treatment and Training Issues.

[b21-99-102] Ries RK (1994). Assessment and Treatment of Patients with Coexisting Mental Illness and Alcohol and Other Drug Abuse. Treatment Improvement Protocol (TIP) Series 9.

[b22-99-102] Watkins KE, Burnam A, Kung FY, Paddock S (2001). A national survey of care for persons with co-occurring mental and substance use disorders. Psychiatric Services.

